# Effects of Per- and Polyfluoroalkyl Substances (PFAS) Exposure Reduction on Serum Lipids in the Copenhagen City Heart Study: A Protocol for an Observational Cohort Study Emulating a Target Trial

**DOI:** 10.3390/toxics14080722

**Published:** 2026-08-14

**Authors:** Georges Khoury, Laura Deen, Lars Christian Lund, Erich Batzella, Michelle C. Turner, Gorm Boje Jensen, Tina Kold Jensen, Sandra Søgaard Tøttenborg

**Affiliations:** 1 Department of Occupational and Environmental Medicine, Copenhagen University Hospital—Bispebjerg and Frederiksberg, 1014 Copenhagen, Denmark; laura.deen@regionh.dk (L.D.); sandra.soegaard.toettenborg@regionh.dk (S.S.T.); 2 Department of Public Health, University of Copenhagen, 1014 Copenhagen, Denmark; 3 Clinical Pharmacology, Pharmacy and Environmental Medicine, Department of Public Health, University of Southern Denmark, 5230 Odense, Denmark; lclund@health.sdu.dk (L.C.L.); batzella@health.sdu.dk (E.B.); tkjensen@health.sdu.dk (T.K.J.); 4 Barcelona Institute for Global Health (ISGlobal), Barcelona, Spain; michelle.turner@isglobal.org; 5 Universitat Pompeu Fabra (UPF), Barcelona, Spain; 6 CIBER Epidemiología y Salud Pública (CIBERESP), Madrid, Spain; 7 Copenhagen City Heart Study, Department of Occupational and Environmental Medicine, Bispebjerg Hospital, Copenhagen, Denmark; gorm.boje.jensen.01@regionh.dk; 8 Department of Public Health, Section of Environmental Health, University of Copenhagen, 1014 Copenhagen, Denmark

**Keywords:** target trial emulation, causal inference, serum lipids, cardiometabolic health, PFAS, adults, cohort

## Abstract

Per- and polyfluoroalkyl substances (PFAS) are environmental contaminants associated with higher serum lipids, although primarily in cross-sectional studies, limiting causal inference. We aim to determine whether there is a causal relationship between PFAS and serum lipid levels by emulating a target trial of a hypothetical PFAS-reduction intervention using observational data. The target trial would enroll adults aged ≥20 years without prior cardiovascular, kidney, or liver disease, diabetes, or use of related medications. Participants would be randomly assigned to either PFAS-reduction counseling or no counseling, with adherence evaluated after 10 years, and then followed up after 10 years to assess effects on serum lipids. To emulate the target trial, we will use data from the Copenhagen City Heart Study on three successive clinical visits: baseline, follow-up, and outcome assessment visit, 10 years apart. Eligible participants meet the target trial criteria, have available blood samples for PFAS quantification at follow-up, and information on serum lipids at the outcome assessment. Intervention strategies will be evaluated based on observed reductions in PFAS concentrations between baseline and follow-up visit. Serum lipids are assessed at the outcome assessment visit. The emulation assumes exchangeability by adjusting for baseline and time-varying confounders using G-computation. This protocol explores applying the target trial emulation framework to improve causal inference in environmental epidemiology.

## 1. Introduction

Per- and polyfluoroalkyl substances (PFAS) are human-made pollutants that persist in the environment and the human body due to their strong chemical stability, bioaccumulation, and long biological half-lives [1,2,3,4]. Numerous epidemiological studies have reported associations between PFAS exposure and higher total cholesterol and low-density lipoprotein (LDL) cholesterol [5,6,7], suggesting a role of PFAS in the pathogenesis of cardiometabolic disease, the leading cause of death worldwide [8].

The proposed biological mechanisms underlying these associations involve several complex pathways that are still under investigation [9]. Treatment of human hepatocytes with PFOA and PFOS downregulated hepatocyte nuclear factor 4-alpha, which in turn repressed cholesterol 7 alpha-hydroxylase, the rate-limiting enzyme responsible for the hepatic conversion of cholesterol into bile acids, leading to cholesterol retention [10]. Another proposed mechanism includes activation of peroxisome proliferator-activated receptors in animal models, which regulate lipid and glucose metabolism, leading to higher serum lipid levels [11].

According to the European Food Safety Authority, causal relationships between PFAS and cholesterol have not been established; however, it was concluded that there is clear evidence for an association between exposure to PFOS, PFOA, and PFNA and increased serum levels of cholesterol [12]. The key reason why a causal association has not yet been confirmed or refuted is that most observational studies investigating the topic have been cross-sectional [12], with minimal or no temporal separation between PFAS exposure and the outcome of interest, raising concerns about reverse causation. Even studies that are longitudinal in data collection often measure change in PFAS and change in outcomes concurrently, preventing clear temporality and rendering them effectively cross-sectional in design [13,14,15,16,17,18,19]. Moreover, because PFAS exposure begins in utero and bioaccumulates throughout life [20], no true onset of exposure can be defined with a single time-point exposure to separate the health effects of current exposure levels from those resulting from the existing body burden.

Overcoming these persistent design limitations is essential for drawing reliable inference on the effects of PFAS on serum lipids. Therefore, we aim to assess whether there is a causal relationship between PFAS and serum lipid levels by emulating a target trial of a PFAS-reduction intervention using observational data from the Copenhagen City Heart Study (CCHS). This protocol specifies the target trial and its emulation.

## 2. Materials and Methods

In this target trial, the population of interest comprises adults, 20 years or older, with no history of cardiometabolic disease who would be randomized to receive PFAS-reducing counseling or no counseling, with adherence evaluated after 10 years to allow sufficient time for natural PFAS elimination and isolate the effect of the PFAS-reducing counseling from residual baseline PFAS levels. Serum lipids would be assessed 20 years post-baseline. We will emulate the target trial using the CCHS cohort by applying identical eligibility criteria and further restricting the cohort to individuals who participated in both the follow-up and outcome assessment visits. Reduced exposure to and increased elimination of PFAS can be achieved through individual PFAS-reducing counseling focused on dietary and lifestyle changes and PFAS awareness [21,22,23]. Since no counseling intervention was conducted in the CCHS, the intervention assignment in the emulation is inferred from changes in serum PFAS concentrations between baseline and follow-up visit, a period during which PFAS is expected to decrease uniformly across our population due to regulatory changes. The emulation therefore relies on an as-treated approach, assuming covariate-adjusted exchangeability, with outcomes assessed 20 years after baseline. In the following, we specify the target trial and target trial emulation according to the Transparent Reporting of Observational Studies Emulating a Target Trial (TARGET) [24]. Table 1 summarizes the target trial and emulation.

### 2.1. Target Trial Specification

#### 2.1.1. Eligibility Criteria

Individuals 20 years or older from Copenhagen, Denmark, would be invited via e-mail to a study visit at the hospital. Participants would be excluded prior to intervention assignment based on health examination screening that identifies a diagnosis of cardiovascular, kidney, or liver disease, diabetes, or current use of medication for any of these conditions.

#### 2.1.2. Intervention Strategies and Assignment

The intervention strategy would consist of PFAS-reducing counseling through lifestyle changes and PFAS awareness, including actionable guidance on informed choices related to food, water, household items, and everyday products, with emphasis on non-PFAS alternatives. The comparison strategy would be no counseling. Eligible participants would be randomly assigned to intervention strategies in a 1:1 ratio, without blinding participants or investigators.

#### 2.1.3. Outcome and Follow-Up Period

**Figure 1:**
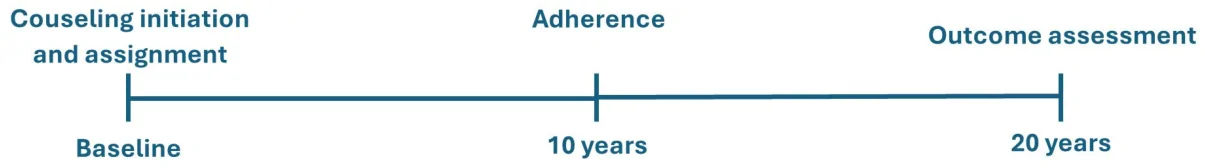
Temporal structure of the target trial.

Outcomes of interest are serum lipid biomarkers including total cholesterol, LDL cholesterol, high-density lipoprotein (HDL) cholesterol, remnant cholesterol, and triglycerides. Follow-up would start at assignment to an intervention strategy at baseline. Loss to follow-up would include nonparticipation in the 20-year outcome biomarker assessment visit, emigration, withdrawal from the study or revocation of consent, or death. Adherence would be evaluated through telephone interview 10 years after intervention assignment. To isolate the effect of PFAS-reducing counseling from residual baseline PFAS levels, it is necessary to allow sufficient time for natural PFAS elimination to occur. For example, Perfluorohexanesulfonic acid (PFHxS) has an estimated biological half-life of approximately 5.3 years [25]. This implies that roughly 75% of baseline serum PFHxS would be naturally eliminated after two half-lives, with even greater elimination expected for the other PFAS with shorter half-lives. To obtain optimal temporal separation between adherence and the outcome, we would wait 10 years to capture long-term effects, effectively separating past exposure from current effects. The temporal structure of the target trial is presented in Figure 1.

#### 2.1.4. Causal Contrast of Interest

The causal contrasts of interest would be the intention-to-treat (ITT) effect and per-protocol (PP) effect. The ITT analysis evaluates the effect of assignment to PFAS-reducing counseling, whereas the PP analysis assesses the effects of counseling among adherent participants, accounting for censoring upon non-adherence to the assigned treatment. Non-adherence would include failure to attend scheduled counseling sessions, premature withdrawal from the intervention, receiving counseling outside the prescribed format or frequency, or seeking counseling independently despite being assigned to the control group. For the ITT analysis, the estimand would be defined as the absolute difference in mean serum lipids after 20 years of follow-up between participants randomized to PFAS-reducing counseling and those randomized to no counseling. For the PP analysis, the estimand would be the absolute difference in mean serum lipids after 20 years, comparing adherent participants in the PFAS-reducing counseling group with adherent participants in the no-counseling group.

#### 2.1.5. Assumptions

For the ITT estimand, we would assume that randomization successfully balances baseline characteristics between intervention arms. We would assume no interference between PFAS-reducing counseling and no-counseling groups, and within each group, i.e., the outcome depends only on their own assignment and not on the assignment of others. We would assume that loss to follow-up is either negligible or occurs at similar rates across the intervention arms (i.e., non-differential with respect to intervention assignment) and that loss to follow-up is independent of the outcome conditional on health status.

For the PP estimand, we would assume no interference between participants. We would assume that adherence to the intervention is measured without error, and that, conditional on measured baseline and time-varying covariates, adherence is independent of the serum lipid outcomes.

#### 2.1.6. Analysis Plan

For the ITT analysis, participants would be analyzed according to their assigned intervention group. The primary analysis would use a linear regression model with the different serum lipids at the outcome assessment visits regressed on the intervention status. The intervention effect would be reported as the adjusted mean difference in serum lipids between randomized groups, with corresponding 95% confidence intervals.

For the PP analysis, non-adherent participants at 10 years would be censored. The PP effect would be estimated using g-computation to adjust for non-adherence with inverse probability of censoring weights (IPCW) to account for administrative censoring due to loss to follow-up. Effects would be reported as adjusted mean differences in serum lipids 20 years after randomization.

### 2.2. Target Trial Emulation

#### 2.2.1. Data Sources

**Figure 2:**
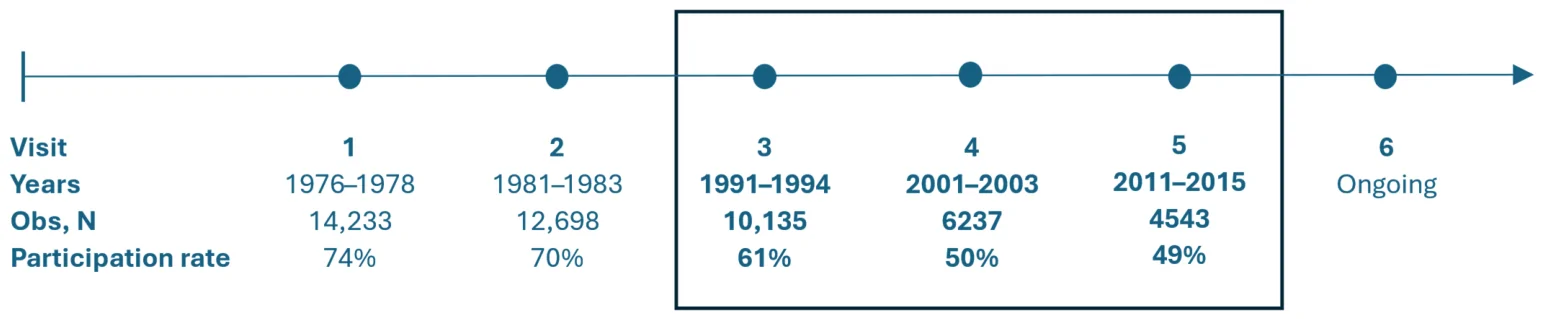
Visits in the Copenhagen City Heart Study.

The CCHS is a large prospective cohort initiated in 1976 to investigate the etiology and prevention of cardiovascular disease and later expanded to include a broader range of health outcomes. Six data collection cycles have been conducted to date, each comprising health examinations and questionnaires in a random population sample living in the eastern part of Copenhagen. The specific area was chosen due to its proximity to the examination site at Rigshospitalet and because it was expected to encompass all socioeconomic groups. Previous participants were re-invited at each subsequent visit. Blood was collected at each examination; some were analyzed for clinical biomarkers, including serum lipids, and some were stored in the Herlev Hospital biobank for future research. Further details about the CCHS are provided in their cohort profile [26]. For the emulation, we will use data from visits 3, 4, and 5 of the CCHS due to the availability of rich covariates (Figure 2).

#### 2.2.2. Eligibility Criteria

The trial emulation will be based on participants who were newly enrolled at visit 3 and participated in visits 4 and 5, which corresponds to the baseline, follow-up, and outcome assessment visits of the target trial. Eligible participants were 20 years or older at baseline, free of self-reported cardiovascular, kidney, and liver disease, diabetes, and reported no use of related medication. Participants will additionally be required to have blood samples from the baseline and follow-up visits available for PFAS quantification and to have data on serum lipid measurements at the outcome assessment visit. This conditioning on repeated participation is necessary because PFAS analyses are costly (approximately €135 per blood sample) and will be implemented to ensure that resources are allocated to participants contributing complete outcome data. The potential selection bias introduced by this design choice is addressed in the methodological considerations.

#### 2.2.3. Intervention Strategies and Assignment

Because no formal PFAS counseling intervention was provided in the CCHS, intervention assignment will be inferred from the observed change in serum PFAS concentrations between the baseline and follow-up visit. The change in each individual PFAS will be analyzed separately. Lower decreases in serum PFAS levels can result from routine laboratory measurement error (assay coefficient of variation, CV) rather than a true physiological change. To avoid misclassifying analytical noise as a genuine decrease, we applied a reference-change-value approach adapted from Harris and Yasaka’s framework [27]. In the primary analysis, for each participant, the laboratory-reported analytical CVs for baseline and follow-up PFAS measurements will be combined, yielding a participant-specific threshold that reflects the total measurement error expected for that individual. Increases or reductions at or below this threshold will be categorized as “increase/no true reduction”; reductions exceeding it will be categorized as “reduction”, representing a true decrease beyond measurement variability. In the secondary analysis, we will divide the “reduction” group by the median to separate lower and higher reductions. This classification aligns with an as-treated analysis, in which participants are analyzed according to their observed exposure change rather than a randomized assignment.

We will measure the following PFAS at the baseline and follow-up visit: Trifluoromethanesulfonic acid (TFMS), Perfluoropropanoic acid (PFPrA), Perfluorobutanoic acid (PFBA), Perfluorobutanesulfonic acid (PFBS), Perfluoropentanoic acid (PFPeA), Perfluoropentanesulfonic acid (PFPeS), Perfluorohexanoic acid (PFHxA), Perfluorohexanesulfonic acid (PFHxS), Perfluoroheptanesulfonic acid (PFHpS), Perfluorooctanoic acid (PFOA), Perfluorooctanesulfonic acid (PFOS), Perfluorononanoic acid (PFNA), Perfluorodecanoic acid (PFDA), Perfluorodecanesulfonic acid (PFDS), Perfluoroundecanoic acid (PFUnDA), Perfluorododecanoic acid (PFDoA), Perfluorotridecanoic acid (PFTrDA), Perfluorotetradecanoic acid (PFTeDA) using on-line solid phase extraction followed by liquid chromatography and triple quadropole mass spectrometry (LC–MS/MS) at Environmental Medicine, University of Southern Denmark. Our criterion for inclusion of individual PFAS exposures in the statistical analyses will be concentrations above the limit of quantification (LOQ) in >75% of samples. Values below LOQ will be substituted with LOQ/2 in the statistical analyses. Quality control and quality assessment will be based on certified reference material (NIST1958) from the National Institute of Standards and Technology and on participation in the external quality control program, German External Quality Assessment Scheme (G-EQUAS) for analysis in biological materials organized by the German Society of Occupational Medicine.

#### 2.2.4. Outcome and Follow-Up Period

Outcomes will be the same as in the target trial. Total cholesterol, HDL cholesterol, and triglycerides were measured in non-fasting plasma using standard enzymatic hospital assays (Boehringer Mannheim GmbH, Mannheim, Germany; Konelab, Thermo Fisher Scientific, Waltham, MA, USA). HDL cholesterol was quantified after precipitation of apolipoprotein B-containing lipoproteins. LDL cholesterol was calculated using the Friedewald equation when triglyceride concentrations were ≤4 mmol/L (≤352 mg/dL) and was measured directly at higher triglyceride levels. Remnant cholesterol was derived as total cholesterol minus the sum of HDL cholesterol and LDL cholesterol.

**Figure 3:**
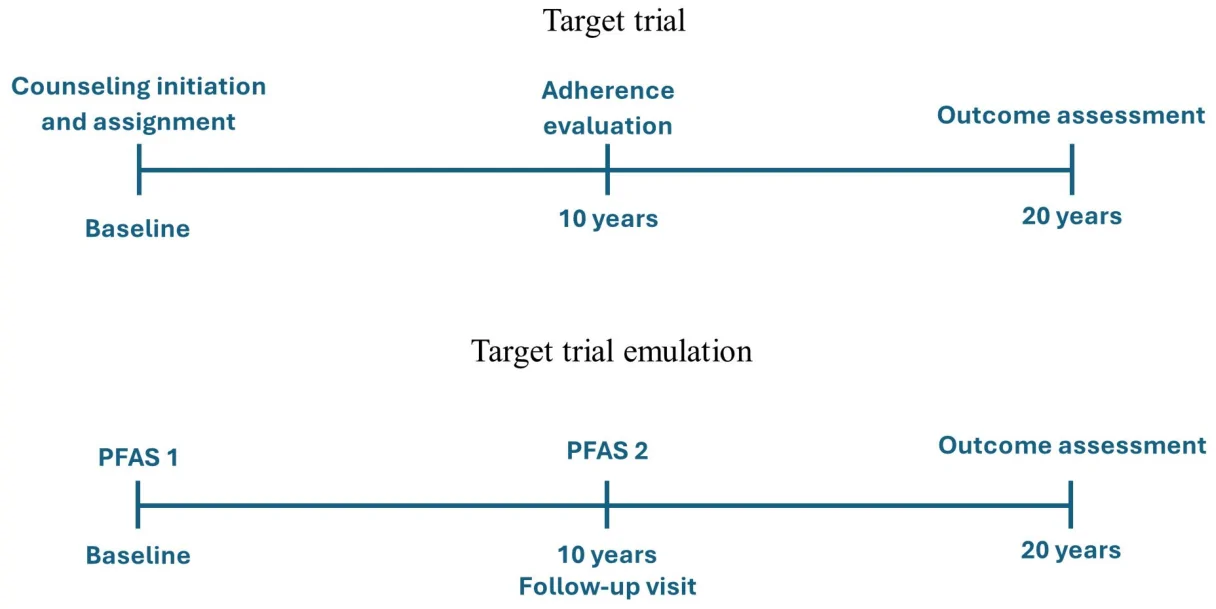
Temporal structure of the target trial and target trial emulation.

Intervention strategies become observable at the follow-up visit (approximately 10 years after baseline, as data collection cycles in the CCHS were not conducted at fixed intervals and varied between participants, with a median interval of 10 years). Participants are subsequently assessed for serum lipids at the outcome assessment visit, approximately 10 years later, effectively 20 years after baseline (Figure 3). Because treatment classification is based on the change in PFAS concentrations between the baseline and follow-up visit, the emulation conditions require continuous participation across these visits; therefore, adherence is complete by design.

#### 2.2.5. Causal Contrast of Interest

Because the intervention assignment is discernible only at the follow-up visit and, by design, everyone is fully adherent, we can estimate as-treated effects.

#### 2.2.6. Assumptions

**Figure 4:**
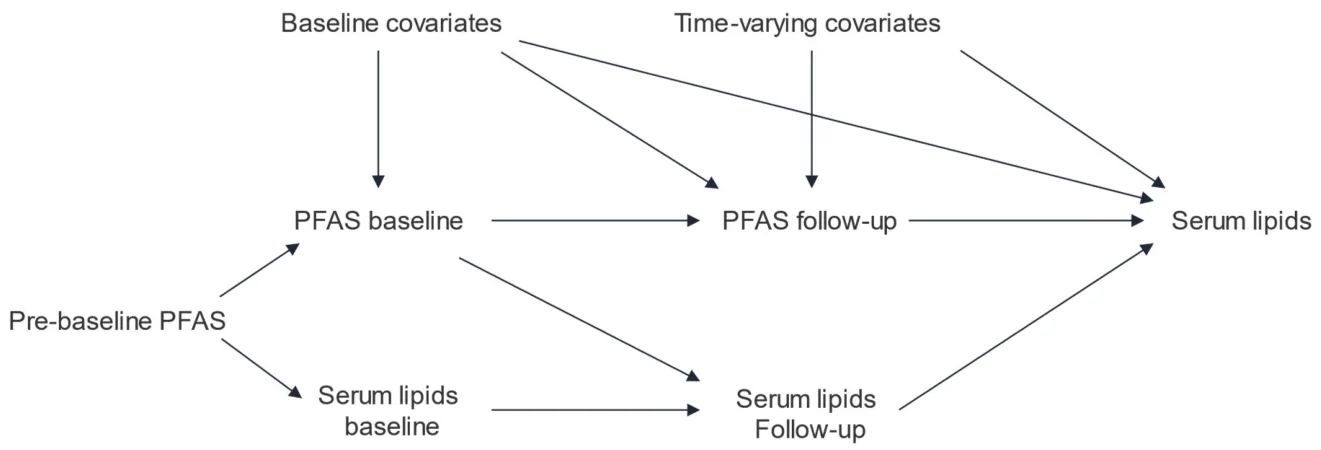
DAG illustrating selection of covariates in the longitudinal causal relationship between PFAS level change over 10 years and serum lipids.

We will assume conditional exchangeability between intervention groups after adjustment for baseline and time-varying covariates. Covariates are selected a priori based on directed acyclic graphs (DAG) (Figure 4). Baseline covariates will be obtained from the CCHS questionnaire and include age (continuous), sex (binary categorical), smoking status (binary categorical), physical activity (hours/week, categorical), alcohol intake (number of standard drinks of beer, wine, spirits; continuous), and family history of cardiovascular disease, diabetes, hypertension, and hypercholesterolemia (binary precision variable). From the baseline clinical examination, we will obtain continuous measures of waist–hip ratio and serum lipid levels (precision variable). Register-based time-varying covariates for highest attained education (categorical) and income (continuous) will be obtained from Statistics Denmark, using data updated annually in the registers for the period between each participant’s baseline and follow-up visits. We will also adjust for baseline PFAS (continuous) if the correlation between baseline PFAS and the change is moderate (<0.8). All continuous covariates will be modeled using restricted cubic splines with 3 knots at the 10th, 50th, and 90th percentiles. We will assume temporality, with PFAS reduction occurring prior to serum lipids assessment and no reverse causation. We will assume consistency; that is, if two individuals exhibit the same measured reduction in PFAS, whether this results from increased excretion or from lower PFAS influx combined with natural excretion, they are considered to have experienced the same exposure for the purpose of the emulation. We will assume positivity, such that within strata defined by measured covariates, there is at least one participant in each PFAS-reduction group, allowing meaningful comparisons across exposure groups. As PFAS will be measured by the same accredited lab in one batch, we will assume that PFAS concentrations are measured accurately and comparably across visits. Because inclusion requires survival and participation in three visits over a 20-year period, a necessary assumption will be that, conditional on the above covariates, loss to follow-up is independent of potential serum lipid outcomes. This assumption is addressed in the methodological considerations section.

#### 2.2.7. Analysis Plan

Distributions of the population characteristics at baseline will be examined by medians, percentiles, and proportions. All percentiles and proportions will be reported in line with national data protection rules, which require that estimates be based on data from at least five individuals [28].

G-computation will be used to estimate the causal effect on 20-year outcomes, comparing the increase-or-no-true-reduction group against the true-reduction group in the primary analysis, and against the reduction group split by the median into lower and higher reduction in the secondary analysis. We will interpret results by evaluating the magnitude, direction, and precision of effect estimates rather than relying solely on binary significance testing, consistent with recommendations from authoritative sources [29]. The outcome model will include baseline covariates as well as time-varying covariates described in the 2.2.6 assumption paragraph. Predicted potential outcomes under each intervention strategy will be generated from the fitted model, and standardized estimates of the mean potential outcomes for each group will be obtained. Average treatment effect will be estimated, and 95% confidence intervals computed via the delta method using the “marginaleffects” package [30] in R 4.5.3.

#### 2.2.8. Sensitivity Analysis

First, because initiating lipid-lowering medication after baseline influences serum lipid outcomes, we will perform a sensitivity analysis in which time-varying lipid-lowering medication use is set to zero. With this, we will simulate a counterfactual scenario in which no participant initiated treatment during follow-up, allowing us to assess how post-baseline lipid-lowering medication uptake influences our effect estimates. Second, to evaluate the implications of using alternative exposure definitions applied in prior PFAS studies, we will estimate the associations between (i) baseline PFAS concentrations and serum lipid levels at the follow-up and outcome assessment visits and (ii) follow-up PFAS concentrations and lipid outcomes at the outcome assessment visit. If estimates obtained using these alternative single time-point exposure definitions differ from those derived from the target trial emulation, such discrepancies would indicate that the conventional observational approach, even when temporality is respected, may still produce biased effect estimates.

## 3. Expected Study Population

**Figure 5:**
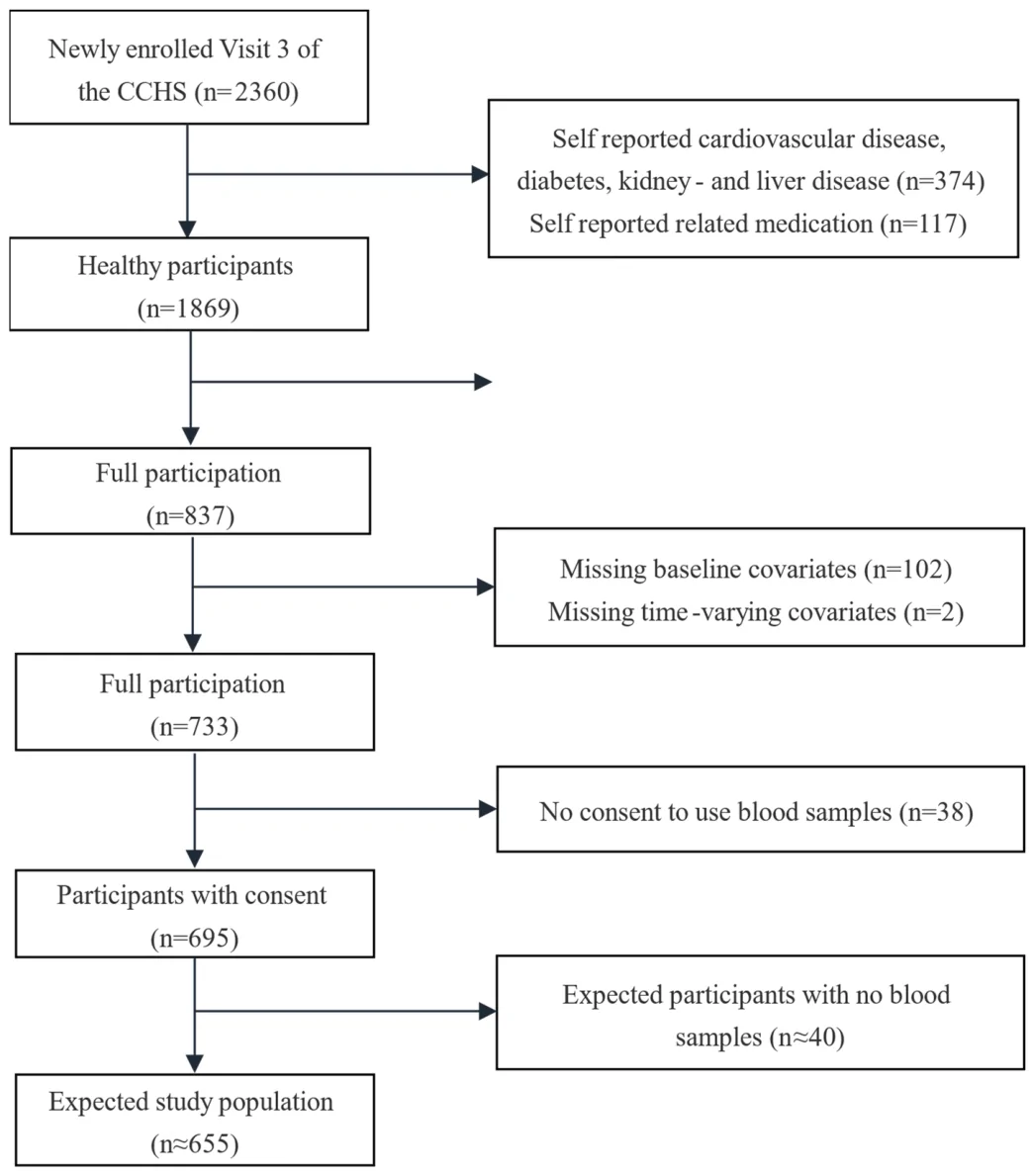
Flow chart of the expected study population for the target trial emulation of the PFAS reduction and serum lipids in the CCHS.

Among the 2360 participants aged ≥20 years who were newly enrolled at Visit 3 of the CCHS, 374 reported cardiovascular, kidney, and liver disease, or diabetes, and an additional 117 participants reported use of related medicine, leaving 1869 healthy participants at baseline. Of these, 728 did not participate in the follow-up visit, and 304 did not participate in the outcome assessment visit, resulting in 837 individuals with full participation. 102 had one or more missing baseline covariates, and 2 had missing time-varying covariates, leaving a final study population of 733 participants. Of these, 38 participants did not provide consent for the use of their blood samples, leaving 695 participants potentially eligible for blood-based analyses. We expect that approximately 40 of the potentially eligible participants will have missing or insufficient blood samples from at least one of the two visits required for PFAS measurement and therefore will be excluded from the final study sample. A flow chart can be seen in Figure 5.

## 4. Methodological Considerations

In an attempt to move beyond conventional exposure-outcome analyses, we explicitly followed the TARGET Statement [24] to articulate a well-defined causal question and to align eligibility criteria, intervention strategies based on observed reduction in PFAS, outcome timing, follow-up, and causal contrasts with a hypothetical PFAS counseling trial. This framework allowed us to transparently document the design choices and assumptions required to emulate such a trial using long-term data from the CCHS.

The CCHS will allow us to leverage temporally separated measurements of PFAS concentrations, with the serum lipids outcome assessed at a subsequent visit, thereby ensuring temporality between exposure and outcome. The 10-year interval between PFAS measurements improves the ability to distinguish new exposure from residual baseline levels. Consequently, the change between the two PFAS measurements more strongly reflects PFAS exposure during the follow-up period and is therefore more informative for evaluating the causal relationship with lipid levels measured 10 years later. In addition, the study will benefit from rich longitudinal covariate information collected through questionnaires and clinical examinations from the CCHS, as well as from national registries, which will support the exchangeability assumption through adjustment for all identified confounders. We will also capture a period during which PFAS exposures in the general population are expected to have declined following regulatory changes, with some compounds showing steeper decreases than others [31].

Serum lipid concentrations were measured in the non-fasting state in the CCHS, consistent with current Danish and broader European laboratory practice. Non-fasting blood sampling does not affect cholesterol, which differs minimally between fasting and non-fasting states [32]. However, triglycerides are known to rise postprandially; therefore, non-fasting measurements introduce additional within-person variability related to unmeasured time since last meal [32]. Because fasting status was uniform across the cohort and unrelated to exposure status, this measurement variability is expected to be non-differential with respect to exposure and to attenuate precision rather than bias the estimated effect.

Baseline diet may be a potential confounder, as dietary habits would affect both PFAS and serum lipid levels [33], and since dietary intake was not measured at baseline in the CCHS, it may challenge the exchangeability assumption. We will adjust for baseline PFAS levels, waist–hip ratio, highest attained education, income, and cholesterol, which will also serve as proxies for baseline dietary habits. While this is true under a classical epidemiological approach with a single time-point exposure, diet poses a particular challenge beyond simple confounding when looking at PFAS change as our exposure. Behavioral dietary change (e.g., reduced consumption of animal-based foods) is part of the causal pathway through which PFAS exposure changes. Adjusting for diet change or diet as a time-varying covariate would result in conditioning on a variable that created the PFAS change itself and would therefore result in removing part of the true causal effect of PFAS exposure reduction on lipid outcomes [33]. In the emulation, we will not be able to observe and distinguish the specific pathways of PFAS reduction. This challenges the consistency assumption, because the health effect of a given PFAS reduction may differ depending on the underlying pathway. For example, reductions driven by dietary changes (e.g., lower intake of animal-based foods) could influence serum lipids through nutritional pathways independent of PFAS. In contrast, reductions due to environmental regulation would not involve these pathways. Consequently, any observed association between PFAS reduction and serum lipids may partly reflect concurrent lifestyle changes, potentially leading to an overestimation of the true effect of PFAS reduction. However, the most plausible driver of reduction in PFAS in this setting is regulatory change during the study period, which would affect exposure uniformly across the population [31]; thus, the consistency assumption likely holds.

**Figure 6:**
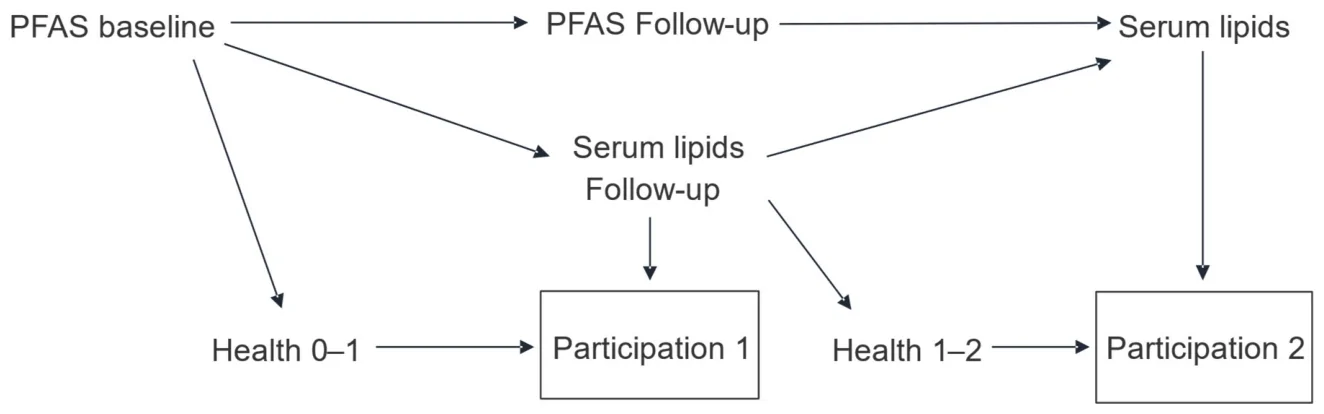
Flow chart of the expected study population for the target trial emulation of the PFAS reduction and serum lipids in the CCHS. *Health 0*–*1 indicates health status of the participants between the baseline visit and the follow-up visit, and Health 1*–*2 indicates health status of the participants between the follow*-*up visit and the outcome assessment visit*. *Participation 1 indicates participation at the follow*-*up visit, and participation 2 indicates participation at the outcome assessment visit*. *A box around a variable denotes conditioning on that variable*.

The emulation conditions require continuous participation across all three visits as well as available serum for PFAS measurement and serum lipid data. If continuous participation is partly related to underlying health status and PFAS simultaneously influence this through their effect on health, this could lead to selection bias. Although inverse probability weighing could address this form of administrative selection, it would require costly PFAS quantification in participants with incomplete follow-up to construct censoring models. This presents a bias-variance tradeoff where the reduction in bias is outweighed by the loss of statistical precision and increased estimator variance. Given these constraints, the design choice of the emulation differs from the target trial and limits our ability to fully emulate the trial’s follow-up process. PFAS was not part of the original scope nor measured during active data collection in the CCHS, and participants were unaware of their PFAS levels at that time (PFAS will be measured from stored blood samples), minimizing the risk of self-selection due to PFAS. As illustrated by the selection-bias DAG (Figure 6), conditioning on participation may introduce selection bias, potentially inducing an association between PFAS changes and serum lipid outcomes even in the absence of a true causal effect. If participants with increases in PFAS concentrations remain in the study, they are more likely to represent a “healthier subset” with more favorable serum lipid profiles compared to the target population, potentially leading to an underestimation of the true effect of PFAS reduction. Accordingly, estimates should be interpreted as conditional on continuous study participation and may not be fully generalizable to the original target population. To assess the magnitude of this selection, we consider comparing baseline PFAS concentrations between individuals in the study population and a random sample of those who participated only at baseline, as well as at baseline and at follow-up, to get an indication of how strongly PFAS is associated with continuous participation. Based on an expected study population of 655, we would need to measure baseline PFAS for 155 individuals in each group to be able to detect a standardized mean difference in Cohen’s d = 0.25 (one-fourth of a standard deviation) using a two-sided alpha of 0.05 and 80% power. A minimum of 87 individuals would need to be measured to ensure detection of a difference no larger than Cohen’s d = 0.32 (one-third of a standard deviation). However, because PFAS testing is expensive, analyzing the samples from these additional individuals is an optional secondary analysis that will only be conducted if additional funding is secured.

We will conduct a complete-case analysis whereby participants with missing baseline or time-varying covariate data (n = 104) will be excluded. Multiple imputation was considered as an alternative approach to handling this missingness. However, imputing the missing data would require an additional costly 208 PFAS analyses (104 participants × 2 [measurements/timepoints]). To assess whether this cost could reasonably be avoided, we conducted a preliminary comparison between the 104 participants expected to be excluded due to missing covariate data and our expected study population, without accounting for additional participants who may be excluded due to missing blood samples (if any) once data collection is complete. The results of this comparison were reassuring: only small differences were observed in income, education, and smoking status between the two groups. This pattern suggests that missingness is consistent with a missing-at-random mechanism. As income, education, and smoking status are included as covariates in the analytic model, complete-case analysis is expected to yield approximately unbiased estimates under this assumption.

The CCHS draws from the general population and includes participants across all socioeconomic categories, supporting the broader relevance of our findings across social strata. The median age of our study population was 37 years, indicating a middle-aged adult population, which should be considered when comparing our results to studies conducted in younger or older cohorts. However, our strict exclusion and inclusion criteria, including exclusion of individuals with different diseases and medication at baseline, ensure that our analytic sample represents a relatively healthy population. As such, our findings may not directly generalize to individuals with pre-existing cardiometabolic disease or treatment, and should be interpreted as most applicable to healthy, middle-aged adults. Geographic location can also shape co-pollutant exposure profiles, a common source of variability in environmental exposure studies, which could potentially limit the generalizability of the results.

## 5. Perspective and Dissemination

Given the increasing public and regulatory attention on PFAS, understanding whether there is a causal relationship between PFAS and serum lipid levels is of critical importance. This protocol focuses on serum lipids, but the framework can also be applied to other cardiometabolic markers, such as liver and kidney damage, as well as inflammatory and diabetes biomarkers. Results will be disseminated through publication in peer-reviewed journals and presentations at national and international conferences. Beyond the immediate findings, this protocol contributes to the scientific community by demonstrating how target trial emulation can strengthen causal inference in environmental epidemiology, where randomized trials are typically not possible.

## Figures and Tables

**Table 1:** Target trial protocol for an observational study using the CCHS cohort to estimate the causal effect of PFAS exposure on serum lipids in adults from Copenhagen, Denmark.

	Trial Specification	Emulation
Inclusion	Age ≥ 20 years.	Same as target trial. Newly enrolled in visit 3 (baseline visit) of the CCHS.
Exclusion	Cardiovascular disease, diabetes, kidney and liver disease based on health examination, or use of medication for any of these conditions.	Same as target trial, but self-reported.
Intervention	Receive counseling for reduction in PFAS through lifestyle changes.	PFAS-related lifestyle changes will be inferred from PFAS measurements at baseline and follow-up visit (change between the two visits). Intervention discernible at follow-up visit. Levels of the intervention: increase or no true reduction in PFAS, and reduction in PFAS. For the secondary analysis, the reduction group will be split by the median into lower- and higher-reduction categories.
Assignment	Randomization 1:1, non-blinded.	As-treated analysis. Assume exchangeability between intervention groups conditional on baseline covariates and time-varying covariates.
Follow-up	Starts at baseline (day of randomization). Censoring at adherence and outcome assessment visit upon non-participation, emigration, death, or 20 years after baseline.	Same as target trial. Non-participants at follow-up or outcome assessment visits will be excluded.
Outcome	Serum lipid biomarkers at 20 years post-baseline.	Same as target trial.
Contrast	Intention-to-treat and per protocol with evaluation of effect of counseling and censoring upon non-adherence evaluated after 10 years.	As-treated only. A follow-up visit is needed to discern intervention (non-discernible intervention strategies), and all individuals are fully adherent to the intervention they receive.

## Data Availability

The authors do not have permission to share the data. Access to data may be granted upon formal request to the CCHS steering committee through Anders Marcuslund-Reuss (anders.marcuslund-reuss.01@regionh.dk). Linkage to register data requires separate applications to Statistics Denmark.

## Abbreviations

The following abbreviations are used in this manuscript:

CCHS	Copenhagen City Heart Study
CV	Coefficient of variation
HDL	High-density lipoprotein
ITT	Intention-to-treat
LDL	Low-density lipoprotein
PFAS	Per- and polyfluoroalkyl substances
PFBA	Perfluorobutanoic acid
PFBS	Perfluorobutanesulfonic acid
PFDA	Perfluorodecanoic acid
PFDoA	Perfluorododecanoic acid
PFDS	Perfluorodecanesulfonic acid
PFHpS	Perfluoroheptanesulfonic acid
PFHxA	Perfluorohexanoic acid
PFHxS	Perfluorohexanesulfonic acid
PFNA	Perfluorononanoic acid
PFOA	Perfluorooctanoic acid
PFOS	Perfluorooctanesulfonic acid
PFPeA	Perfluoropentanoic acid
PFPeS	Perfluoropentanesulfonic acid
PFPrA	Perfluoropropanoic acid
PFTeDA	Perfluorotetradecanoic acid
PFTrDA	Perfluorotridecanoic acid
PFUnDA	Perfluoroundecanoic acid
PP	Per-protocol
TARGET	Transparent Reporting of Observational Studies Emulating a Target Trial
TFMS	Trifluoromethanesulfonic acid
